# Creating a Cleaned Dataset for Ease of Use

**DOI:** 10.23889/ijpds.v9i5.2775

**Published:** 2024-09-18

**Authors:** Saira Ahmed, Daniel S Thayer, Muhammad A Elmessary, Ieuan Scanlon, Etienne Badoche

**Affiliations:** 1 Swansea University

## Objective

The Welsh Longitudinal General Practice (WLGP) dataset contains over 4 billion records. Due to the size and the need to filter the dataset to get the necessary general practice interaction results, query performance is poor. To overcome this, a ‘cleaned’ version of the dataset needed to be created.

## Approach

An R package was created that would be run when a new refresh of the WLGP data is provided. Two new tables would be created based on the original WLGP Events table.

## Results

A WLGP Cleaned Dataset R Package was produced and creates a reformatted events table and events look up table. The reformatted GP events table reduces the original events table from 14 columns to 8 key columns. The events look up table takes distinct events from the original WLGP events table and produces a new table including columns such as the event description, type and hierarchy levels. Both tables are then linked using an event code ID.

## Conclusion

The R package now creates a new reformatted events table as well as an events look up table which is run after a WLGP refresh is provided. The new tables are then provisioned to any projects that have access to the dataset.

## Implications

The new tables has improved query performance for our researchers, providing them with a table with all the necessary information and an easy way to decipher events codes. This has improved the amount of time researchers have spent manipulating and querying the tables.

